# Detection of *Wuchereria bancrofti* in human blood samples and mosquitoes in Matayos, Busia County-Kenya

**DOI:** 10.1038/s41598-023-46329-z

**Published:** 2023-11-08

**Authors:** Nancy Kinyatta, Dorcas Wachira, Rosemary Githae, Japheth Lusweti, Johnstone Ingonga, Christine Ichugu, Caroline Maina, Rukiya Haji, Francis Kimani, Rael Musili, Jacinta Muli, Luna Kamau

**Affiliations:** 1 Kenya Medical Research Institute, Centre for Biotechnology Research and Development, P.O Box 54840-00200, Nairobi, Kenya; 2 Department of Bioinformatics and Molecular Biology, Jomo Kenyatta University of Agriculture and Technology, P.O Box 62000-00200, Nairobi, Kenya

**Keywords:** Diseases, Medical research

## Abstract

Lymphatic filariasis is a mosquito borne disease which leads to abnormal painful enlarged body parts, severe disability and social stigma. We screened *Wuchereria bancrofti* in Matayos constituency in Busia County. Blood samples were collected from 23 villages selected purposively based on clinical case reports. Finger prick and/or venous blood sampling and mosquito collections was carried out. Antigenaemia and filarial DNA prevalence were determined. Infection rates on mosquito pools were estimated and SPSS version 26 was used for descriptive statistics analysis. A total of 262 participants were recruited, 73.3% (n = 192) of the participants had no symptoms, 14.1% (n = 5.3) had swollen legs, 5.3% (n = 14) had painful legs and 3.8% (n = 10) with scrotal swellings. Average antigenemia prevalence was 35.9% (n = 94) and DNA prevalence was at 8.0% (n = 21). A total of 1305 mosquitoes were collected and pooled into 2–20 mosquitoes of the same species and from the same village. Two pools out of 78 were positive for filarial DNA with a minimum infection rate of 0.15%. From this study, antigenaemia and infected mosquitoes are an indication of active transmission**.** The clinical signs are evidence that filarial infections have been in circulation for over 10 years. The global climate change phenomenon currently happening has been shown to adversely affect the transmission of vector borne diseases and is likely to increase lymphatic filariasis transmission in the area. This study therefore recommends further screening before Mass Drug Administration, morbidity management and enhanced mosquito control Programmes are recommended in the study area.

## Introduction

Lymphatic filariasis (LF), commonly termed as elephantiasis, is a mosquito borne neglected tropical disease. Infections occur when filarial parasites of *Wuchereria* or *Brugia* genera are transmitted to humans by mosquitoes during blood sucking^1^. Climate changes have greatly influenced vector borne disease transmission as vectors occur in regions where they were not initially documented or increase in numbers due to different rainfall pattens and temperature changes. This has resulted to vector borne disease transmissions in areas normally considered as non-endemic^2^. Vectors have also developed adaptability mechanisms to drastic climatic changes making them maintain their vectorial capacity to various disease transmissions. They also become more resistant to common insecticides hence difficult to control them. *Wuchereria bancrofti* is the most common causing 90% of all the lymphatic filariasis infections worldwide. Infections are usually acquired in childhood and is the second cause of chronic global deformity that impairs the lymphatics, leading to long-lasting disability^3^. Chronic manifestations of the disease include adeno-lymphangitis, lymphedema, elephantiasis and genital deformities^3^. In 2020, 863 million people in 50 countries were living in areas that required preventive chemotherapy to stop the spread of the infections. Globally, 36 million people remain with chronic disease manifestations of which 25 million men have hydrocele and over 15 million people with lymphoedema^1^.

Lymphatic filariasis affect the poor and marginalized communities in tropical and sub-tropical regions. It is a major cause of morbidity, severely affecting socio-economic development in the endemic regions^4,5^. The disease contributed to 5.25 million disability- adjusted life years (DALYs) in 2002, which dropped to 2 million DALYs by the year 2016^6^. Individuals suffering from lymphatic filariasis experience repeated filarial attacks known as adenolymphangitis (ADL), which hinder them from actively participating in social and economic activities^7,8^. The economic burden due to filariasis in the US alone amounts to $5.765 billion annually^9^ of which $114.69 is associated to the chronic manifestations and acute attacks of the disease ^9,10^. Genital damage in men causes social stigmatization and severe handicap leading to physical limitations which results to 83% of the total economic loss due to the fact that young adults contribute more to economic development^11^. In Africa, lymphatic filariasis causes almost US $1 billion loss each year^12^. The morbidity also causes a huge burden to individuals, households, government-funded and private healthcare systems^13,14^. Surgical management of hydrocele causes strain to already overburdened health-delivery systems^15^. Lymphedema of the lower limbs and genital parts enlargement is shame and taboos in women and are considered undesirable. Affected persons feel severely stigmatized and marriages in many situations become unstable with separations or even impossible for the youths^16^. School going children fail to attend schools due to morbidity and sometimes leading to school drop-outs^12^.

In the year 1997, The World Health Assembly (WHA) Resolution 50.29 called for elimination of lymphatic filariasis as a public health problem. In response, WHO initiated the Global Programme to Eliminate Lymphatic Filariasis (GPELF) by the year 2020, currently the elimination is targeted to be achieved by 2030^6,15^. The GPELF proposed a comprehensive elimination strategy, including (i) Mass drug administration (MDA) to entire at-risk population for transmission interruption in endemic communities (ii) Morbidity Management and Disability Prevention (MMDP) strategy to alleviate suffering. Parasite transmission interruption can be achieved with MDA coverage at 80–90% in the intervention units for over 5 to 6 years or longer in areas with high baseline microfilaria (mf) prevalence^3^. Annual MDA reduces the density of parasites in circulating blood of the infected people to levels where transmissions can no longer be sustained by mosquito vectors^17^. For MDA interventions, single dose of 2 anthelminthic drug combinations is administered: albendazole (ALB) (400 mg) + diethylcarbamazine (DEC) citrate (6 mg/kg); ALB (400 mg) + ivermectin (IVM) (150–200 µg/kg) or in combination of the above 3 drugs (ALB + DEC + IVM) to all at risk population in endemic regions^17^. There has been significant progress in the control of lymphatic filariasis by the GPELF with over 8.6 billion cumulative treatments delivered to stop the spread of infection since the start of the Program in 2000^17^. Reports have shown that, in many endemic countries such as Ghana, there are still pockets of hotspots even after 10 or more rounds of treatment^18^. Global estimation of people requiring MDA dropped from 1.4 billion in 2011 to 492 million in 2020 due to successful implementation of WHO strategies with 24 lymphatic filariasis endemic countries (of 83) not requiring MDA and were conducting post-MDA surveillance ^18–20^. To achieve the second goal of GPELF, a core strategy of Morbidity Management and Disability Prevention (MMDP) is needed. Suffering caused by the disease can be alleviated through a minimum recommended package of care to manage chronic stages; lymphedema, elephantiasis and hydrocele. These services should be made available in primary health care systems in all endemic regions^21^.

Lymphatic filariasis has been documented in Kenya since 1910^22^. Comprehensive mapping of the disease in Kenya was done between 1998 and 1999 during which only coastal region was found to have prevalence that could be termed as endemic according to WHO guidelines^1,6, 23^. To date, the disease remains endemic in coastal regions of Kwale, Mombasa, Malindi, Kilifi, Tana River, Lamu and Taita Taveta^23^. About 2.5 million people in Kenya are at risk of infection^24,25^. All control efforts have been directed and scaled up only in the regions where the disease has been found to be endemic^25^. Kenya, an endemic country, launched its National Programme for Elimination of Lymphatic Filariasis (NPELF) in 2002 in Kilifi District which was extended to Malindi and Kwale Districts in 2003. By 2011, only four rounds of MDA had been given in some districts with significant decrease in microfilaria prevalence of 15–25% in 2006 to 1.8–7.6% in 2022 and the antigenemia prevalence of 35% decreased to 6.3% in the same years^26,27^ , while other districts such as Tana River had received only one round^24,28^. The lymphatic filariasis MDA programs in Kenya are under the Division of Vector-Borne and Neglected Tropical Diseases, (DVB-NTD) in the Ministry of Health and the implementation is guided by the Kenya NTD breaking transmission strategy, 2019–2023. The plan is to expand MDA coverage, implement Water, Sanitation, and Hygiene (WASH) interventions and implement Behavior Change Communication (BCC) as a comprehensive package^29^. There is need for the endemic countries which are about to reach the elimination target to do a comprehensive assessment for re-mapping, monitoring and evaluating the impact control of the programs in place.

In addition to the documented endemic regions in Kenya, there are however strong speculations of lymphatic filariasis circulations in other regions including Lake Victoria region particularly in Kisii, Kisumu and Western Kenya Counties. Recently, presence of circulating filarial antigens were detected in patients around Lake Victoria region (N. Kinyatta, unpublished data) indicating that other regions of the country are at risk of the disease.

The western part of Kenya is susceptible to filarial infections due to its proximity to Lake Victoria and Uganda which is lymphatic filariasis endemic area. In addition, the climate of this region favors mosquito vectors capable of transmitting filariasis including *Culex sp, Anopheles sp*, *Aedes sp*. A number of lymphatic filariasis infection cases have been reported in Busia and some referred to KEMRI-Alupe for diagnosis (Busia referral hospital records; N. Kinyatta, personal communication). World Health Organization recommends that a prevalence of more than 1% filarial antigenaemia and 2% microfilaremia be considered endemic^6^. Kenya as well as many other endemic countries had not attained a prevalence threshold to be declared free from lymphatic filarial infections by the year 2020 as earlier proposed^24,25^. Thus, more MDA rounds and scaling up to other susceptible regions was recommended. Kenya’s endemicity stands at 1.8–7.6% in different coastal areas though MDA has been going on since 2002^27^. One of the challenges was that, MDA in Kenya had not been conducted annually between the years 2002–2014 as recommended due to financial constraints. However as from 2015, MDA has been taking place annually in selected regions and the introduction of three drug regime (Albendazole 400 mg, Ivermectin 200 mg and Diethylcarbamazine citrate 6 mg/kg body weight) in Kenya in 2020 have enhanced transmission control^24,25^. Population coverage of above 80% was also attained in these regions and this has accelerated the control target^25^. However, several gaps exist that need to be bridged before the National programme for elimination of lymphatic filariasis (NPELF) declares Kenya free from filarial infections. For instance, mass screening of other suspected regions needs to be carried out and have NPELF extended to the affected regions. In this study, we screened lymphatic filariasis in Busia County in Kenya through human and mosquito infections assessment so as to provide information to what is already documented for filariasis from other at-risk regions. The study thus generates critical data on infections in the region that will advise policy makers, Ministry of Health, NPELF and other stakeholders on the need to embark on the necessary control efforts in the region with the overall goal of moving closer to Kenya being declared free from lymphatic filariasis infections.

## Materials and methods

### Study site

**Figure 1:**
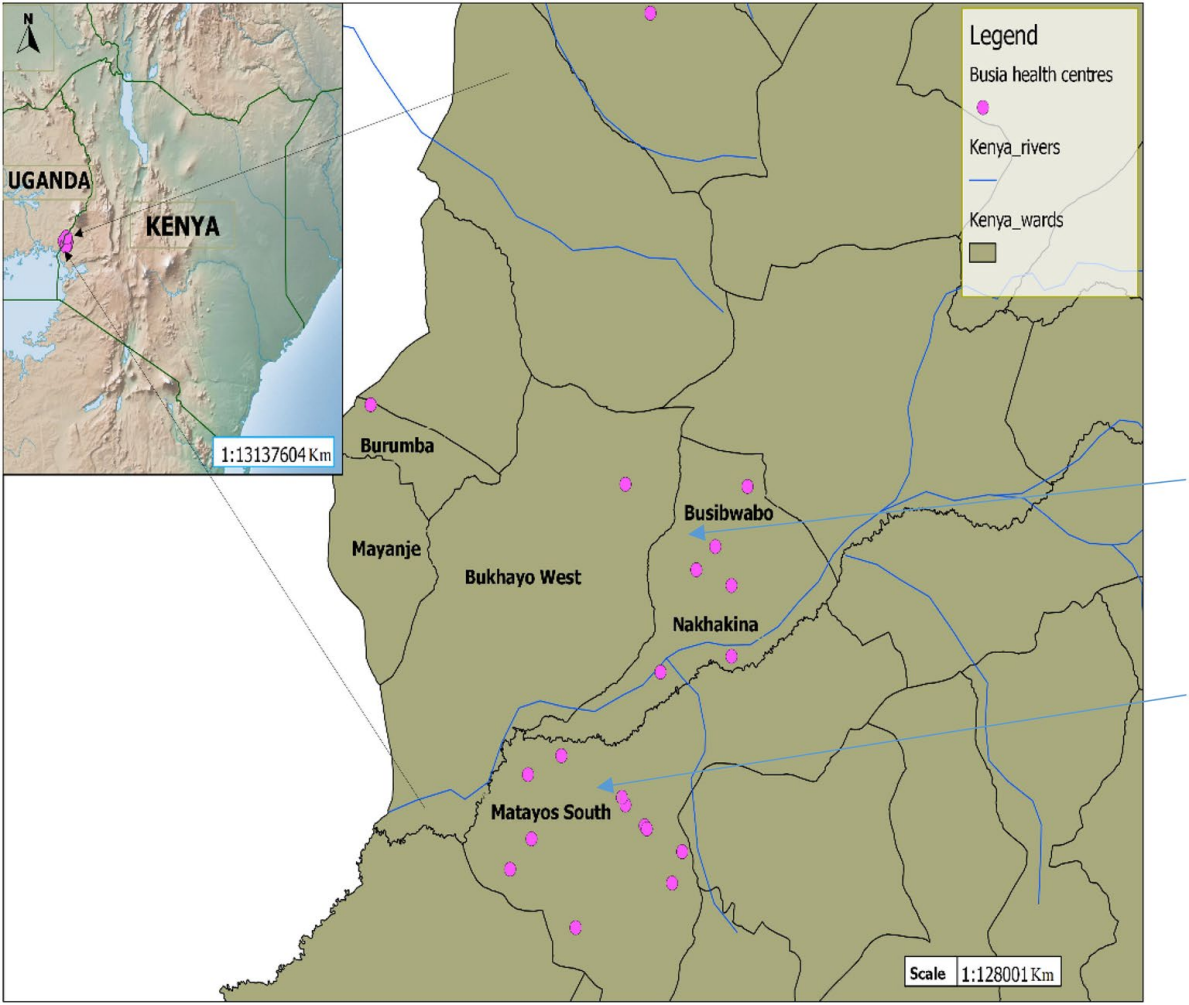
Map of Kenya showing Busia County; Matayos south and Busibwabo wards as the study sites pointed by blue arrows. Source: Map generated with QGIS software program using the Matayos study site Co-ordinates taken during the study, courtesy of Jacob Mueti.

This study was carried out in Matayos Constituency, Busia County. Matayos South and Busibwabo wards were selected for the study because cases of filarial-like lymphedemas had previously been reported in these areas (N. Kinyatta; Personal observations) and there were no lymphatic filariasis control Programmes going on in this region. Busia County (00 27 11N, 34 07 30E) is located in the western part of Kenya approximately 268 miles (431KM) west of Nairobi and east of Busia District in Uganda. The 2012 population census estimated the population of Busia to be 816,452. The main economic activity in the region is fishing and agriculture. The area has a tropical humid climate due to the influence of Lake Victoria with average annual temperatures of between 24 and 26 °C and temperatures ranging between 17 and 30 °C. The mean annual rainfall in Busia ranges between 900 and 1500 mm distributed throughout the year. The long rains are usually experienced between March and June with short rains falling between September and October. The two wards were purposively selected to participate in the survey-based study on the presence of chronic cases of the disease and/or environmental factors indicating that lymphatic filariasis transmission is likely to occur in the region as given in the WHO-AFRO guidelines for mapping of lymphatic filariasis^30^. Figure 1 below shows the study site where blood samples and mosquitoes were collected from.

### Study design

The study design was both case and cross-sectional community-based survey that aimed at mapping lymphatic filariasis in Matayos constituency, Busia County.

### Survey strategy

The lymphatic filariasis survey was conducted using house-to-house approach by a team of two scientists, three laboratory technicians and a clinician. Additionally, a village elder and a local Community Health Volunteer (CHV) in each selected village joined the survey team to assist with mobilization of community members and give directions of the area.

### Clinical examination of participants

Inclusion criterion for participants was the presence of chronic clinical signs of lymphatic filariasis which included lymphedema, hydrocele and elephantiasis and those living closely to the affected individuals particularly members in the same homestead. Lymphedema were defined as swellings due to fluid accumulation as a result of lymphatic system blockages by filarial worms and hydrocele refers to scrotal swelling due to filarial infections. Elephantiasis is hardening and thickening of victim’s skin due to bacterial and fungal infections^3^. Individuals above 2 years of age were legible for the study and only consenting adults and children whose parents had given consent for the children’s participation in the study were recruited, with children above 7 years of age giving assent. A total of 23 villages in the study area fitting this criterion were identified for sampling.

### Blood samples collection

Human blood samples were collected from recruited study participants from their homesteads between June, 2019 and March, 2020 during the day (0800 h–1800 h EAT). To test for the presence of circulating filarial antigen (CFA), approximately 75 μl of blood was obtained from finger prick and tested using Alere Filarial Test Strips (FTS). Mass drug administration with Albendazole 400 mg was done to all community members by the clinician as one of antifilarial drugs used for clearing microfilariae and deworming other intestinal helminths. For CFA positive individuals, an additional 4 ml of blood was drawn through venipuncture of the median cubital vein for parasite characterization using conventional Polymerase Chain Reaction (cPCR) and sequencing assays.

### Mosquito collection

Mosquitoes were collected both indoor and outdoor from 6.00 pm to 6.00 am using CDC Light traps from the selected homesteads of CFA-positive participants^31^. For the indoors, the traps were set about 1 m from the beds or sleeping places to maximize on the chances of getting fed-infected mosquitoes. This study used CDC light traps only, outdoor traps were set near windows and doors to increase the chances of getting mosquitoes entering the houses in search of blood meal. Geographic Information System (GIS) coordinates of the sampling sites were recorded and QGIS software used in developing map of the study area for other follow up studies.

### Sample processing

Blood samples were tested for circulating filarial antigen using Filarial Test Strip (FTS) in the field. Seventy-five micro liters (75 μl) of blood sample was taken by finger-prick, then slowly added onto Alere™ Filariasis Test Strip (FTS) (Alere©, Waltham, United States), following user instructions^32^. Briefly, the test cards were placed on a table and left for 10 min before reading the results. For CFA positive samples, a second 75 μl blood sample obtained by finger-prick was collected for a confirmatory test as described above. For positive cases, 4 ml blood was collected by venipuncture into vacutainers and taken to the Kenya Medical Research Institute (KEMRI) laboratories in Nairobi for further analysis by DNA extraction and PCR assays. Mosquitoes sampled were identified morphologically using entomological key^33^, and 10% of daily collection immediately dissected in search of microfilariae. Mosquitoes were preserved under silica gel for transportation to KEMRI for DNA analysis. Mosquitoes were pooled according to same species and the area of collection, each pool contained 2–20 mosquitoes. Deoxyribonucleic acid (DNA) extraction was done in the pools using DNeasy blood and tissue kit (Qiagen, German) following Manufacturer’s manual and microfilaria DNA detected by conventional PCR assays^34^.

### Detection of *W. bancrofti* by polymerase chain reaction assays

#### Deoxyribonucleic acid (DNA) amplification

Deoxyribonucleic acid (DNA) extraction from human blood was done as described by Abbasi and others with minor modifications^34^. PCR targeting the Ssp1 repeat sequence was then carried out using the extracted DNA in a PCR thermocycler using primer sequences NV1-5′ CGTGATGGCATAAAGTAGCG 3′ and NV2-5′ CAACCAGAATACCATTCATCC 3′ identified by Zhong et al*.*^35^*.*

#### PCR product analysis

PCR product size fragmentation was done in a 2% (W/V) agarose gel, which was prepared by dissolving 3.0 g of molecular grade agarose (Sigma) in 150 ml of 1X TAE buffer and casted on gel tank to polymerase for 30 min. Two microliters (2 µl) of loading buffer (Promega) were added to 5 µl of the amplified product of each sample and loaded in the wells. The gel was run in a horizontal electrophoresis tank (Bio Rad^®^). DNA amplified products were visualized as bands under ultraviolet (UV) light on a transilluminator, the gels photographed using digital camera and the results recorded.

#### Scoring of the bands

The positive bands in the gel electrophoresis were recorded as those appearing at the position equivalent to 188 bp (non-coding DNA sequence in *W. bancrofti* Ssp1 repeat DNA sequence) on *W. bancrofti* DNA positive control and the DNA molecular size marker^35^.

#### Data management and processing

The data was entered in data record books and Microsoft Excel spread sheet. Participants’ information was handled with confidentiality and the samples coded with numbers known only to the PI and co-investigators. The data was analyzed using SPSS version 26.0 to obtain descriptive statistics on social demography, correlating signs and symptoms with age groups. The age groups were stratified into 10-year difference per strata. Disease prevalence’s were by PCR and FTS tests. Statistical significance between indoor and outdoor mosquito collection was done by regression. Tables and bar graphs were used on excel spread sheet to present the results.

### Study approval, informed consent and ethical considerations

Approvals for conducting this research were obtained from the Scientific and Ethical Review Unit (SERU), Kenya Medical Research Institute, Protocol Approval No. KEMRI/ SERU 3561. Permission to carry out the study in the county was obtained from Busia County Health Executive.

Informed consents were obtained from the participants after explaining to them the purpose of the study to the participants, for the children below 16 years of age, informed consent was obtained from the parents or guardians. All the study procedures were carried out according to relevant human subject research guidelines^36^. The antigenemia results were given to the study participants individually and given albendazole (400 mg) drugs. Those with lymphedemas were given antifungal cream (Clozole-B 15 g) which contains Beclomethasone and Clotrimazole used in the treatment of infections in the wounds. The patients were advised on general foot hygiene and exercise to reduce swellings. Participants names were not used in the manuscript and consent was obtained for use of their photos taken without showing their faces.

## Results

### Descriptive analysis of study participants

**Figure 2:**
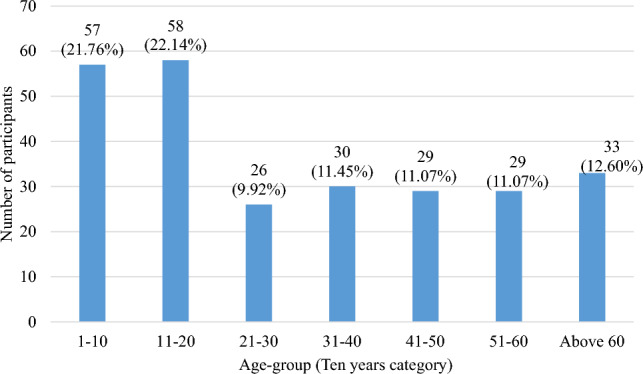
Bar graph showing number of participants per age group.

Differential diagnosis was done on observable chronic cases of lymphatic filariasis (lymphedema, elephantiasis and hydrocele), infection history, detection of circulating filarial antigen test by Filarial Test Strip and filarial DNA detection by PCR assays in human blood and mosquitoes. In this study a total of 262 participants were recruited, with age limit between 4 and 88 years. Participants were grouped by 10-year age difference into 7 categories which were presented in bar graphs in both numbers and percentages. Age group 11–20 years had the highest participants (22.14%) while age group 21–30 had the least number of participants (9.92%). The highest proportion of participants (22.14%) were aged between 11 and 20 years as shown in Fig. 2 below**.** Most of the participants comprised of females (61.07%, n = 160).

Table 1 below shows the number of participants sampled in each village.

**Table 1:** Number of participants sampled in village.

Village	Frequency	Percentage
1 Bulandi	13	4.9
2 Emasiebia	14	5.3
3 Mugweso	57	21.8
4 Sogomere	3	1.1
5 Busende	64	24.4
6 Bundonga	5	1.9
7 Muyafwa	13	8.8
8 Buroboi	1	0.4
9 Murede	23	8.8
10 Nagoma	1	0.4
11 Sirova	6	2.3
12 Lwanya	11	4.2
13 Mavanga	2	0.8
14 Mumbiri	5	1.9
15 Nasiri	8	3.1
16 Bumanyi	4	1.5
17 Bukhanguli	1	0.4
18 Nakhalina	3	1.1
19 Busibwabo	2	0.8
20 Akitesi/Teso	7	2.6
21 Luliba	1	0.4
22 Nang’oma	7	2.6
23 Nang’oma cu	11	4.2
Total	262	100

Busende village had the highest number of participants comprising (24.4%, n = 64) while Buroboi, Nagoma, Bukhanguli and Luliba had the least participants (0.4%, n = 1) as shown in Table 1 above.

### Signs and symptoms presentation in the study participants

Symptoms varied among the study participants. Most of the participants (73.3%, n = 192) had no signs. Participants with swollen legs were 14.1% (n = 37), with painful legs were 5.3% (n = 14), swollen scrotum 3.4% (n = 9) and those with both swollen and painful legs were 3.8% (n = 10) as shown in Table 2 below.

**Table 2:** Signs and symptoms found with the participants in the study area.

Symptoms	Frequency	Percentage (%)
Swollen leg(s)	37	14.1
Painful leg(s)	14	5.3
Swollen scrotum	9	3.4
Swollen and painful leg(s)	10	3.8
No symptoms	192	73.3
Total	262	100

**Figure 3:**
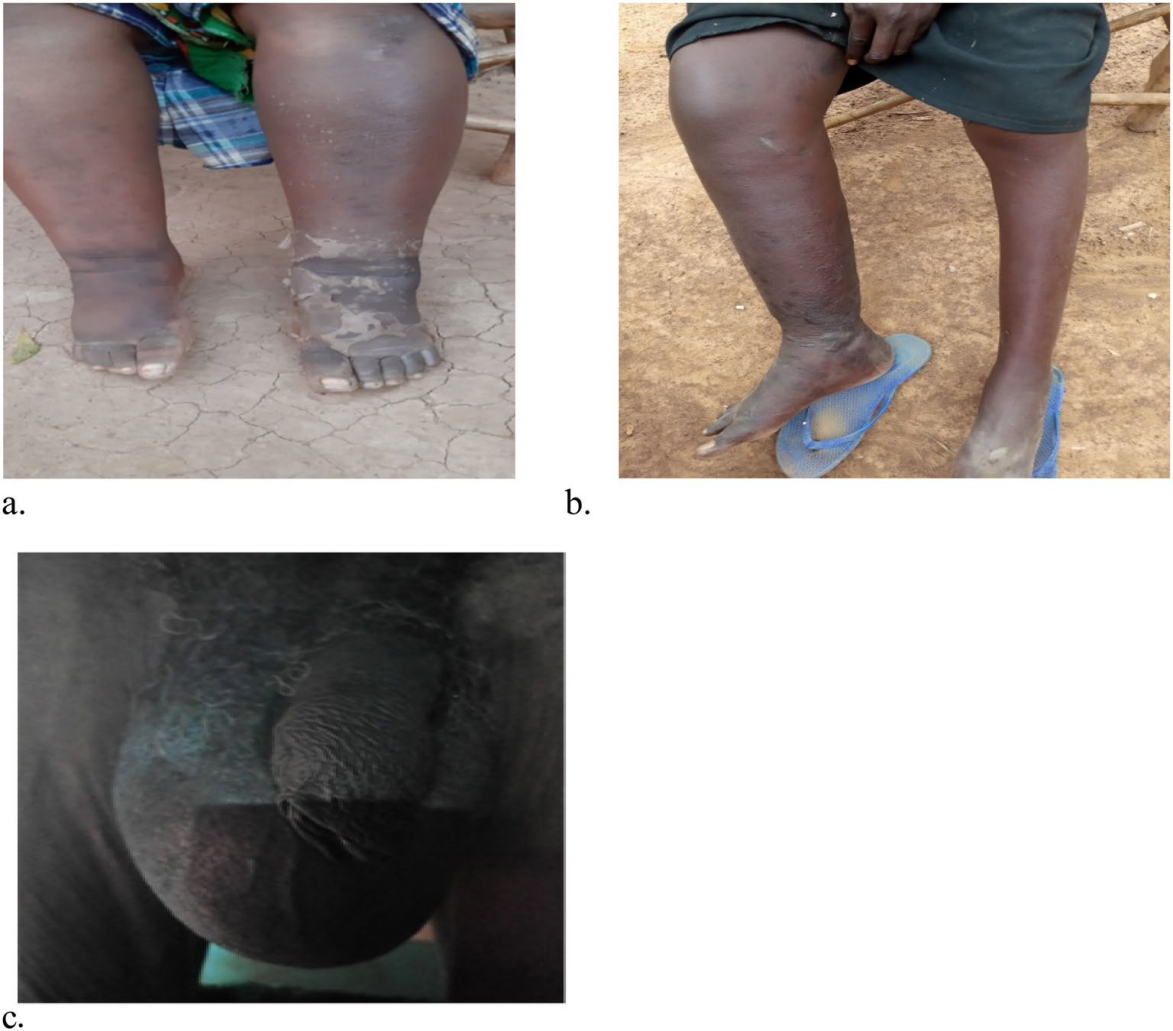
Shows some of the victims with chronic clinical manifestations of lymphatic filariasis disease in the study area. Chronic cases among the study participants; Image (**a**) lymphedema of both legs, images (**b**) lymphedema affecting one leg and image (**c**) scrotal swelling (Hydrocele cases). Source: Photos courtesy of Nancy Kinyatta & Johnstone Ingonga.

Most of the participants found in the study area had no signs, Swollen leg(s) was the common signs found with the participants (Fig. 3).

### Duration of symptoms

**Figure 4:**
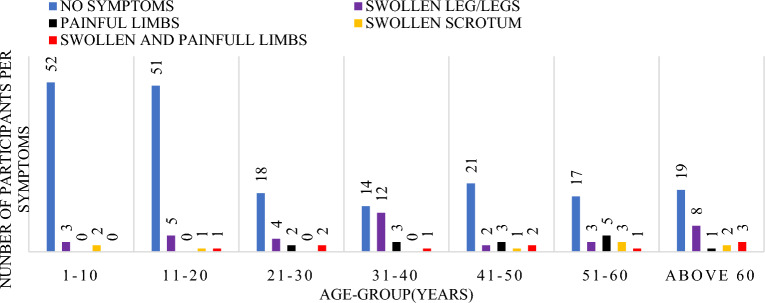
Graph of the signs and symptoms per age group.

The number of years that participants reported having experienced symptoms was analyzed in bands of 10 years shown in Fig. 4 below. A total of n = 29 participants representing 11.1% of the total study participants reported having had swollen leg(s) for a period between 1 and 10 years. Only one participant had both swollen and painful legs and had lived with the symptoms for a period between 21 and 30 years. Other symptoms noted were painful legs for a period of 1–10 years and swollen scrotum for a period above 30 years, however a total of n = 192 (73.3%) participants showed no symptoms of the diseases.

Symptoms distribution within 10 year age-group categories, with majority in each group having no signs. There was a high significance between the duration of symptoms and the age group (df = 6, f = 5.228 Sig. 0.001).

### Prevalence of lymphatic filariasis in the study area

From this study, 94 out of 262 participants tested positive by Filarial Test Strip giving an overall prevalence of 35.9% and 21 participants testing positive for *W. bancrofti* DNA, a prevalence of 8.0%. Participants aged above 60 years had the highest antigenaemia prevalence of 42.9% compared to the other age groups (Table 3) while the least prevalence (22.8%) was found within age group 1–10 years of age. Females also had a higher infection prevalence compared to males (19.5% in females versus 16.4% in males). A number of the participants (24%) showed positive FTS results without any notable symptoms. Among the villages, the highest prevalence was noted in Busende having a prevalence of 7.2% which had also the highest number of participants.

**Table 3:** Filarial infection prevalence per age group in the study population.

Variable	Level	Positive FTS	Positive PCR	Total participants	Prevalence (%) FTS	Prevalence PCR (%)
Age group (years)	1–10	13	2	57	22.8	3.5
	11–20	24	3	58	**41.4**	**5.1**
	21–30	9	0	26	**34.6**	**0**
	31–40	11	5	30	**36.7**	**16.6**
	41–50	12	2	29	**41.4**	**6.8**
	51–60	11	6	29	**37.9**	**20.7**
	Above 60	14	3	33	**42.4**	**9.1**
	Totals	94	21	262		
	Overall prevalence				35.9	8.0

Prevalence values in bold.

**Figure 5:**
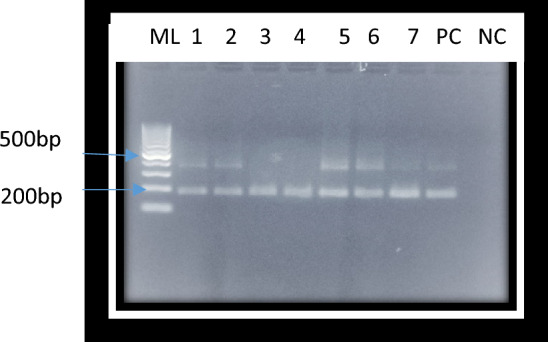
Shows a gel electrophoresis fragmentation of amplified *W. bancrofti* DNA from blood samples and mosquito pools. Image of *W. bancrofti* amplified targeted region of 188 bp on 2% agarose gel electrophoresis. ML is Molecular size standard marker (100 bp), Lanes 1–5 are *W. bancrofti* DNA from blood specimen while lane 6–7 represents *W. bancrofti* DNA from mosquito pools, Lane PC is *W. bancrofti* DNA positive control and Lane NC represents negative control which was deionized PCR water.

Filarial infections in each group were determined by FTS and PCR tests and prevalence for each group calculated. The overall infections for each test were also calculated. The detection of PCR amplified *W. bancrofti* DNA was through gel electrophoresis fragmentation as represented in Fig. 5 below.

### Mosquito species distribution, blood feeding and infection rates.

Indoor and outdoor mosquito trapping using CDC light traps was carried out in five (5) villages in Matayos Busia. A total of 1305 mosquitoes were collected. Indoor collection comprised 96.09% (n = 1254) of all collections while outdoor collection comprised of 3.91% (n = 51) (Table 4).

**Table 4:** Summary of the indoor and outdoor mosquito genera collected.

Mosquito genus	Site of collection		Total
	Indoor count/percentage (%)	Outdoor count/percentage (%)	Genus collected count/percentage (%)
*Anopheles* species	829 (63.52)	3 (0.23)	832 (63.8)
*Culex* species	387 (29.66)	46 (3.52)	433 (33.2)
*Aedes* species	24 (1.84)	1 (0.08)	25 (1.9)
*Coquillettidia* species	14 (1.07)	1 (0.08)	15 (1.1)
Total	1254 (96.09)	51 (3.91)	1305 (100)

The collected mosquitoes belonged to four genera of mosquitoes on morphological identification, namely *Anopheles* species*, Culex* species*, Aedes* species and *Coquilletidia* species*. Anopheles* species were the majority (n = 832, representing 63.75%) of the total collection, while *Coquilletidia* species were the least with 1.15%, n = 15). Majority of mosquitoes collected outdoors were *Culex* species which comprised of 90.2% (46 out of 51 mosquitoes) as shown in Table 4 below.

Mosquitoes were classified as either having taken blood meal (fed) or not taken blood meal (unfed). Out of 1305 mosquitoes collected, majority of them were unfed 87.36% (n = 1140) while fed mosquitoes were 12.64% (n = 165). The source of blood meal was however not determined in this study. There were no filarial larvae found in 131 mosquitoes dissected.

### Mosquito species distribution in each village

**Figure 6:**
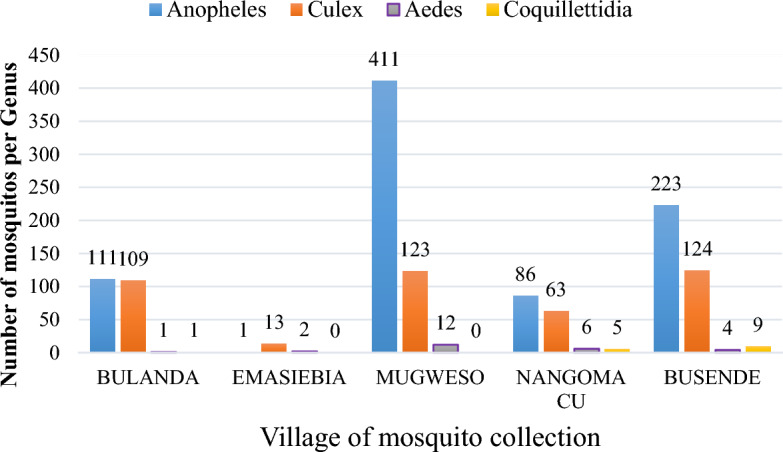
Graph showing mosquito genus collected in each village.

Figure 6 shows mosquito species distribution in the collection area.

Mosquitoes obtained were members of *Anopheles, Culex, Aedes* and *Coquillettidia* species as indicated in Fig. 6 above with Mugweso village having the highest *Anopheles* species caught.

The collected mosquitoes were pooled into 78 pools each pool containing 2–20 mosquitoes of same species collected in the same study village in the same day. Two (2) pools belonging to *Anopheles species* from Mugweso Village tested positive for *W. bancrofti* DNA as in Table 5 below. Minimum Infection Rate (MIR) was estimated by determining the number of infected mosquitoes per 1000 tested given as; [number of positive pools/total specimens tested] × 1000) as per the formula of Biggerstaff^37^. The (MIR) in Matayos was [2/1305]1000 = 1.532.

**Table 5:** Mosquito pooling per genus and village of collection.

Village of collection	Mosquito species								PCR	Total pools
	*Anopheles *species		*Culex *species		*Aedes *species		*Coquillettidia *species			
	Count	Pools	Count	Pools	Count	Pools	Count	Pools		
Bulanda	111	**6**	109	**6**	1	**1**	1	**1**	1	**14**
Emasiebia	1	**1**	13	**1**	2	**1**	0	0	0	**3**
Mugweso	411	**21**	123	**7**	12	**1**	0	0	1	**29**
Nangoma CU	86	**5**	63	**4**	6	**1**	5	**1**	0	**11**
Busende	223	**12**	124	**7**	4	**1**	9	**1**	0	**21**
Total Pools		45		25		5		3	2	**78**

Significant values are in bold.

78 mosquito pools were obtained upon pooling them by each days’ collection as per species and village of collection.

## Discussion

In resource-limited settings such as Africa, disease prevention, diagnosis and management efforts tend to focus on zones with clearly characterized disease transmission patterns. Despite reports on lymphatic filariasis in Busia area of Western Kenya, there have been no definitive studies of prevalence and transmission in the area. This study therefore sought to determine the prevalence of *Wuchereria bancrofti* infections within the population and to determine the presence of vectors of lymphatic filariasis and infection rates with *W. bancrofti* in Matayos Constituency, Busia County (Fig. 1).

Among the villages sampled, Busende Community Unit had the most participants (24.4%) (Table 1) and this could be attributed to the time spent in that unit and the mobilizing ability and awareness created by the Community Health Volunteer (CHV) involved. This can also be associated with the fact that majority of the infected participants were from this villages hence many participants turned out for testing. Majority of people who turned up for screening were in 11–21 years age bracket representing (22.14%) of the screened population (Fig. 2) with (P = 0. 001). This age group was found to be the most enthusiastic to participate in the study being the first one of its kind in the study population, these observations were similar to the findings of Kagai and colleagues in 2008 at the Kenyan coast^38^. In addition, this is the age group recommended by WHO for *W. bancrofti* disease surveillance. In this study, this group had antigenaemia prevalence of 41.4% though majority of them had no symptoms. Infections take long period of time to manifest into chronic stages and thus victims remain asymptomatic for many months or even years but remain major carriers and transmitters of circulating microfilaria. The disease manifests into chronic stages with time if individuals in asymptomatic group are left untreated. Majority of the participants in the study were females and this could be because females in this rural setting are more available and can easily be found at homes compared to men who tend to work away from their homes. Also, some men especially those with hydrocele conditions are reluctant to take part in such studies and shun testing due to stigma associated with the disease.

Lymphatic filariasis manifests in asymptomatic and symptomatic forms; including acute and chronic forms. The chronic forms are presented as lymphedemas, elephantiasis or hydrocele (scrotal swellings) cases as shown in Fig. 3. In this study, most of the participants did not show any signs, with 73.3% of the antigenaemia cases being found within the asymptomatic group (Table 2), thus, advocating for mass screening and treatment in this region is crucial. However, due to asymptomatic nature of the disease and its slow progression to chronic stages, some cases are not detectable during mass screening as infected individual may test negative before the parasite multiplies to detectable levels, also majority of patients with chronic conditions test negative for antigens since some do not have circulating filarial antigens because the worms might have died long ago thus not reproducing though the victim has long term deformities.

From our visit to Busia referral hospital, the doctors revealed that there were no records of filarial infections due to lack of diagnostic capacity (Test Kits), it was reported that the suspected cases were usually referred to KEMRI-ALUPE, Busia for diagnosis. On reaching out to some individual cases who were referred to KEMRI-ALUPE, the participants indicated that microscopy of night collected blood was done in search of microfilaria and medications prescribed for them (N. Kinyatta, Personal communication). The participants reported that they would feel better on medications but the swellings would only reduce and never disappear, eventually they would give up on seeking for medical help, therefore swellings would progress to chronic stages with time. In this study, participants with chronic stages of the disease reported that they had lived with the disease even for over thirty years and there was a high significance between the duration of symptoms and the age group (df = 6, f = 5.228 Sig. 0.001). It is unfortunate that victims only seek for medical attention at chronic stages which have no treatments apart from surgical interventions for hydrocele cases. Morbidity and disability management interventions only help relief pain but not to cure the disease.

There was a great discrepancy found in antigenemia (FTS) (35.9% and DNA(PCR) (8.0%) prevalence (Table 3), we associate this to the fact that most FTS antigen test kits lack sensitivity and also detect other related helminths. The obtained prevalence in this study were much higher as compared to the other endemic regions in Coastal Kenya, in which the preverance stands at 1.8–7.6%^27^. This is due to the fact that coastal region in Kenya has been on MDA since 2002 with no MDA in Busia County where this study took place. This study recorded hydrocele prevalence of 3.9%, however Community Health Volunteers reported that there were a lot of hydrocele cases within the age 18 years old and above but most victims did not turn up for screening or failed to disclose their status due to fear of intimidations and stigmatization. It was further reported that some locals believed that the disease is as a result of witchcraft or that it is a hereditary disease (referred to as family disease) resulting in failure to seek medical attention by some victims even when capable of paying for hydrocelectomy procedures. There is therefore need to create more awareness of the disease transmission and manifestations so that the community is aware that elephantiasis is an infectious disease transmitted by mosquitoes and it is preventable and treatable at early stages.

Four mosquito genera were sampled from Matayos-Busia with *Anopheles* species found to be most abundant (Table 4). Busia County in Kenya is known to have high malaria prevalence transmitted by *Anopheles* species of mosquitoes as reported by Edward et al*.*^39^. The rainfall and the climatic conditions are favorable for maintaining vectors breeding sites. Each village had different number of mosquitoes collected suggesting that there were differences in ecological factors in different villages sampled. This was noted as more mosquitoes were obtained from areas near water bodies in accordance to earlier reports from a study by Kinyatta et al. ^40^ in Tana River. A significance different on the mosquito species obtained from the collection villages was found (df = 4, f = 18.163 and p = 0.001). Mugweso village had more mosquitoes with two positive pools belonging to *Anopheles* species, which was also the most abundant species in the village. Mungweso village was found to have a lot of water bodies in the villages as compared to other collection sites. Seasonal variation and mosquito breeding studies by Evans et al. ^41^ showed that mosquito density increases during the wet season due to the availability of mosquito breeding site and favorable climate^2^. Uncontrolled urbanization and poor sanitation amenities have contributed to increase in mosquito breeding sites for vectors of filarial infections^2^. *Culex* species are found to breed in wet pit latrines while *Mansonia* species attach on submerged vegetations for breeding^42,43^. There was a correlation between mosquito genera collected and the site (indoor and outdoor of trappings) (df = 1, f = 56.286 sig. 0.001). Majority of the mosquitoes obtained from indoors were those of *Anopheles* species while those collected outdoors majority were of *Culex species* (Table 4). This is associated with the fact that many *Anopheles* mosquitoes seek for blood meal and rest indoors. The different indoor and outdoor collection in mosquito numbers and species is attributed to the fact that mosquitoes have different host seeking behaviour and resting behaviour.

Filarial infection rates in vectors are important parameter in determining transmission indices. Infection rates refers to the presence of any larvae stages within the mosquito body parts while infectivity rate is the presence of L_3_, which is the infectious stage. Different mosquito species have different capacity of carrying the larvae to infection stages and thus not every mosquito carrying L_1-_ L_2_ has the vectorial potential of transmitting filariasis^44^. The low levels of transmissions of lymphatic filariasis in the vectors is linked with the use of different vector control measures by the community for malaria control programmes. For instance, studies have shown that the use of Insecticide Treated Bed Nets (ITN), which was implemented by the malaria control programmes reduced filariasis transmission in endemic regions of Kilifi and Kwale counties^45^. Importantly, use of LLIN and deworming programs have significantly contributed to reduce lymphatic filariasis infection despite the irregular implementation of MDA as it was found by Njenga et al.^24^ in the coastal regions of Kenya.

From the data gathered, it was evident that filarial infections are a concern in the study areas of Matayos South and Busibwabo wards in Busia due to high number of chronic manifestations found with active transmission being a possibility as a result of the infected mosquito pools and positive *W. bancrofti* DNA detection (Fig. 5). The fact that the disease progression to clinical signs takes many years, the disease remains neglected though it is of a major public health concern. The participants in this study reported that chronic forms of the disease cause a huge social and economic burden to the individuals, relatives and the health care facilities as pointed in other studies by Njomo et al.^45^. Some of the affected participants mentioned having lived with the disease even for over thirty years. This suggested that even during the initial mapping in Kenya (1998–1999)^23^, there may have been a few cases that did not meet the threshold required for the area to be considered an endemic and have control measures put in place. Identification and assessment of these infections haven been inhibited by clinically silent periods before pathognomonic presentations occur in an individual and the lack of testing kits in local hospitals, thus the few case infections resulted to transmission in the population at large and with time, some cases developed to clinical stages.

Climatic changes affect mosquito biting rates, resting, mating behaviors and dispersal resulting to increased vector densities and a raise in disease transmissions indices as it has been reported in other studies ^46–48^. Analysis of long term climate patterns in the study area (Data not shown) revealed an increase of 1.28 °C and 1.35 °C in the mean minimum and maximum temperatures, respectively in the last 60 years, in concordance with predictions that temperature will have risen by 1.0–3.5 °C by 2100 ^49,50^. Lymphatic filariasis transmission in the area is therefore likely to increase in the absence of disease prevention and management measures. There is therefore need for screening of large populations to be able to ascertain the exact extend of the infections in the population to guide decisions on Mass Drug Administration.

## Conclusions

WHO defines lymphatic endemic areas as those with an epidemiological threshold of antigenaemia or microfilaremia equal to or greater than 1% and recommends that control interventions be put in place^1^. Filarial antigenemia prevalence in this study was above this threshold standing at 35.9% and 8% as determined using the FTS and PCR methods respectively. In as much as the inclusion criterion for participants in the current study was the presence of chronic disease or living closely to those with chronic disease, results underscore the need for prioritizing the study area for control and preventive efforts. The chronic cases of lymphedemas and hydrocele which were evident in this study require interventions for Morbidity Management and Disability Prevention (MMDP). Although hydrocele cases can be easily managed through surgical intervention, access to this intervention has been hindered by lack of finances for many. There is also need for enhanced commitment by disease control Programmes within the ministry of health to support prevention and intervention efforts, create wider public awareness as well as integrate management efforts with those for other disease. The findings of this study should motivate epidemiological studies in the larger population for conclusive determination of lymphatic filariasis infection status in Busia County and recommendation of an appropriate action plan.

### Study limitations

The study was carried out within a small population with selection criterion biased to those with chronic diseases manifestations, this cannot really define the exact prevalence in the area and may result to overestimated prevalence’s. In addition, this study did not consider night blood collection for microfilariae detection due to ethical and social cultural beliefs of the participants.

Mosquitos’ collection was done only by CDC light traps and only a few mosquitoes were dissected, different trapping methods needs to be considered for proper mosquito density comparison. The trappings were not done across different seasons hence seasonal comparison was not possible.

Finally, this study aimed at determining presence of *W. bancrofti* infections in the study area and we recommend more studies to be carried out in the study area to ascertain the prevalence for MDA recommendations.

## Data Availability

The data for this study was included in the manuscript text.
